# Intracranial Vertebrobasilar Calcification in Patients with Ischemic Stroke Is a Predictor of Recurrent Stroke, Vascular Disease, and Death: A Case-Control Study

**DOI:** 10.3390/ijerph17062013

**Published:** 2020-03-18

**Authors:** Jožef Magdič, Nino Cmor, Matevž Kaube, Tanja Hojs Fabjan, Larissa Hauer, Johann Sellner, Slaven Pikija

**Affiliations:** 1 Department of Neurology, University Medical Center Maribor, 2000 Maribor, Slovenia; jozef_magdic@yahoo.com (J.M.); tanja.hojs@gmail.com (T.H.F.); 2 Faculty of Medicine, University of Maribor, 2000 Maribor, Slovenia; nino.cmor@gmail.com (N.C.); matevz.kaube@gmail.com (M.K.); 3 Department of Psychiatry, Psychotherapy and Psychosomatic Medicine, Christian Doppler Medical Center, Paracelsus Medical University, 5020 Salzburg, Austria; l.sellner@salk.at; 4 Department of Neurology, Christian Doppler Medical Center, Paracelsus Medical University, 5020 Salzburg, Austria; s.pikija@salk.at; 5 Department of Neurology, Klinikum rechts der Isar, Technische Universität München, 81675 München, Germany; 6 Department of Neurology, Landesklinikum Mistelbach-Gänserndorf, 2130 Mistelbach, Austria

**Keywords:** intracerebral artery calcification, atherosclerosis, ischemic stroke, vascular disease, mortality, prognosis

## Abstract

Intracranial artery calcification can be detected on nonenhanced brain computer tomography (NECT) and is a predictor of early vascular events. Here, we assessed the impact of vertebrobasilar artery calcification (VBC) on the long-term risk for recurrent stroke and vascular events. We performed a case-control trial of all consecutive stroke patients admitted to the University Hospital of Maribor, Slovenia over a period of 14 months. VBC was defined as presence of a hyperdense area within vertebrobasilar arteries that exceeds > 90 Hounsfield units as seen on NECT. Clinical follow-up information was obtained from the hospital documentation system and mortality registry of the district and included recurrent stroke, subsequent vascular events (myocardial infarction, heart failure, peripheral arterial occlusive disease), and death. We followed a total of 448 patients for a median of 1505 days (interquartile range, IQR 188-2479). Evidence for VBC was present in 243 (54.2%) patients. Median age was 76 years, recurrent stroke occurred in 33 (7.4%), any vascular events in 71 (15.8%), and death in 276 (61.6%). VBC was associated with a higher risk of recurrent stroke (hazard ratio, HR 3.13, 95% confidence interval (CI 1.35–7.20)) and vascular events (HR 2.05, 95% CI 1.21–3.47). Advanced age, male gender, and ischemic stroke involving the entire anterior circulation raised the likelihood for death. We conclude that the presence of VBC in patients with ischemic stroke is a short- and long-term prognostic factor for stroke recurrence and subsequent manifestation of acute vascular disease. Further understanding of the pathophysiology of VBC is warranted.

## 1. Introduction

Atherosclerosis is a potentially modifiable driver of overall morbidity and mortality [1]. Arterial calcification is an integral part in the process of atherosclerosis and can be detected in up to 90% of atherosclerotic plaques [2,3]. Radiological presence and extent of calcification is hence used as a noninvasive marker for the burden of atherosclerosis [4].

Intracranial artery calcification (IAC) is a common finding on nonenhanced computer tomography (NECT) of the brain in elderly patients, especially in patients with vascular risk factors [5,6,7,8]. A neuropathological study revealed that there are two distinct patterns of intracranial atherosclerosis based on morphology and potentially pathogenesis [9]. The first pattern is characterized by hardening of the arteries due to accumulation of lipids and calcium in the intimal layer of the vessel. The second variant is specific circular calcification of the internal elastic membrane. Most importantly, these two patterns can also be distinguished on NECT [10].

Several studies have proven that IAC increases the risk for the occurrence of vascular events within 2 years from acute ischemic stroke [11,12,13]. In this regard, both a population and a hospital-based cohort study showed that IAC is linked to a higher rate of stroke, independently of cardiovascular comorbidity [12,14]. There is limited knowledge about IAC of the vertebrobasilar vessels (VBC) and contribution of its presence to stroke recurrence, vascular events, or death [7,8]. Previous studies disclosed a risk for early vascular events, and confirmation on the long-term risk would have significant implications on secondary prophylaxis and subsequent follow-up examination [15]. In this study, we evaluated whether the presence of VBC in patients with ischemic stroke is a short- and long-term prognostic factor for stroke recurrence or subsequent manifestation of acute vascular disease.

## 2. Materials and Methods

### 2.1. Patient Cohort

The local ethics board (UKC-MB-KME-29/19) approved this study. We performed a case-control analysis of all consecutive patients admitted to the University Hospital of Maribor, Slovenia and with confirmed acute ischemic stroke over a period of 14 months from September 2011 to October 2012. Diagnosis of stroke was according to the World Health Organisation (WHO) criteria and the work-up aimed at determining the cause of stroke included brain imaging, electrocardiography (ECG), heart ultrasound, neck vessel ultrasound, and lab examinations. Stroke etiology was classified according to Trial of Org 10172 in Acute Stroke Treatment (TOAST). The subgroups included LAA–large artery atherosclerosis, CE–cardioembolism, SAO–small arterial occlusion (lacunar), UNK–unknown, and UND–undetermined (multiple causality). We also evaluated clinical presentation according to the Oxfordshire Community Stroke Project (OCSP). The subgroups were TACI–total anterior circulation infarction, PACI–partial anterior circulation infarction, LACI–lacunar infarction, and POCI–posterior circulation infarction [16,17]. We excluded patients where no NECT was available and with permanent residency outside of the district.

### 2.2. Evaluation of Intracranial Calcification

All brain images were recorded on a multidetector CT scanner (Aquilion 64, Toshiba Medical Systems, Tochigi, Japan). Specifications were 120 kV and 150–350 mAs (mean value, using automated exposure control) and matrix size of 512 × 512 with mean equivalent dose 1.9 mSV. Slice thickness was 3 mm.

Evaluation of IAC was performed in a CT window with a width of 700 and a length of 250. The Hounsfield Unit (HU) cut-off above 90 was used to detect vascular calcifications. We studied intracranial arteries of the anterior and posterior circulation. Severity of IAC was calculated by using the “calcification” score we reported previously [8]. Briefly, the number of focal calcifications seen in both vertebral arteries and basilar artery (VBC) were summed to define a calcification score. An extra point was added when crescent and/or circular type of calcification was present. We added the total number of slices with calcifications to this score. Thus, the score incorporates both horizontal and vertical dispersion of calcifications and yields an ordinal scale that ranges from 1 to 19 [7,8]. Furthermore, we noted details of the calcification pattern, which included focal, crescent, and circular IAC.

### 2.3. Case-control Study

During the observation period that extended from discharge (if alive) to 1 April 2019, we collected clinical information on recurrent stroke and subsequent vascular events from the hospital information system. This hospital database is monthly updated with information on death cases from the national database. We defined stroke recurrence including transitory ischemic attacks and ischemic stroke when a patient was evaluated within the hospital for sudden onset of neurological symptoms with or without corresponding morphologic correlate on neuroimaging that could not be explained other than ischemic cerebrovascular event. Vascular events included hospitalization due to myocardial infarction, heart failure, and peripheral artery occlusive disease. The causes of death were recorded but not used in analysis. This was related to known low-reliability of exact cause of death and low frequency of clinical autopsies.

The cohort of patients without calcified intracranial vertebral arteries and/or basilar artery (nonVBC) served as the control group.

### 2.4. Statistical Analysis

Due to non-normality distribution of our data we used nonparametric tests (Kruskal–Wallis test) for comparison between categorical and interval variables. Fisher-exact test was used for categorical variables. When testing for association between total vascular events and calcium score, we used analysis of variance (ANOVA) with Tukey’s honestly significant difference (HSD) correction. Cox-proportional hazard model was employed to test for factors that may be associated with stroke recurrence, vascular event, and death due to any cause. We set a P-value below 0.05 as cut-off value for statistical significance. All statistical analysis was performed using R software [18].

## 3. Results

### 3.1. Study Cohort

**Figure 1:**
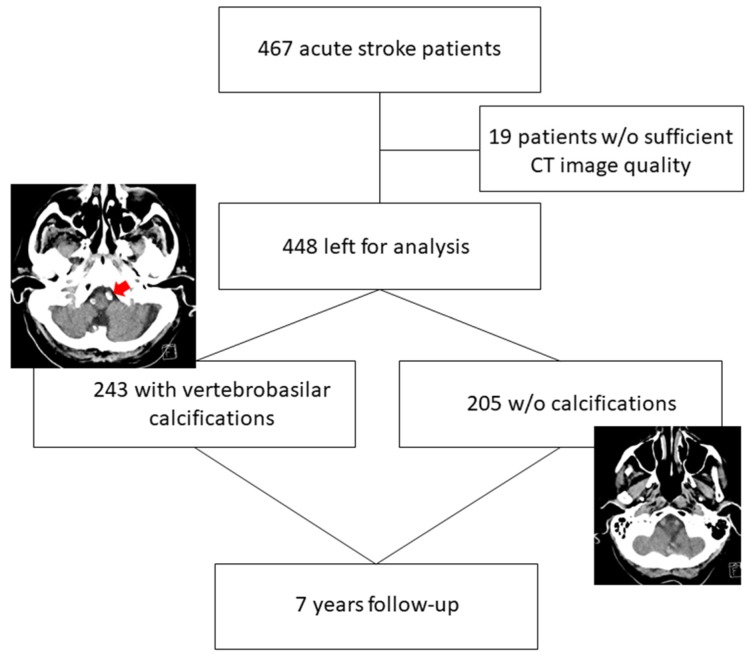
Patient flow diagram during the 7-year study period. Left image: The red arrow depicts calcifications on nonenhanced brain computer tomography (NECT) in left (and right–not labelled) vertebral artery in an 89-year-old man with stroke (representative for the vertebrobasilar artery calcification (VBC) group). Right image: no calcification in vertebrobasilar vessels in an 88-year-old woman with stroke (control group, nonVBC).

There were 467 patients admitted with acute ischemic stroke. Nineteen patients were excluded, the reasons included lack of NECT and insufficient quality. Thus, we enrolled 448 (52.7% women) patients for the case-control study, the work-flow for excluded patients is shown in Figure 1.

Further demographics and disease characteristics of the cohort are shown in Table 1.

### 3.2. Characteristics of Patients with Vertebrobasilar Calcification

We found VBC in 243 (54.2%) patients, and the remaining patients served as the control group (nonVBC). Table 1 compares clinical information for VBC and nonVBC patients.

Crescent and circular calcifications (with or without focal calcifications) were present in 84 (34.5%) patients with VBC. Intracranial carotid artery (ICA) calcifications (any side) were detected in 444 (99.1%). Patients with VBC were more often woman (57.6% vs. 46.8%, *P* = 0.029) and older (78.6 (interquartile range (IQR) (63.4–79.9) vs. 72.4 (63.4–79.9), *P* < 0.001) than those without. Moreover, patients with VBC had more often a history of ischemic stroke (21.8% vs. 12.7%, *P* = 0.013), were less frequently smoking (27.2% vs. 40.0%, *P* = 0.013)), and had a higher rate of atrial fibrillation (29.2% vs. 11.7%, *P* = 0.020). Notably, no details about smoking were available in 20.1% of patients. Patients with VBC had a lower prevalence of small artery occlusive stroke (SAO) etiology according to TOAST criteria (7.8% vs. 13.2%, *P* = 0.017) and lacunar stroke according to OCSP (0.8% vs. 7.3%, *P* = 0.002).

### 3.3. Follow-up and Clinical Outcome

The patients were followed for a median time of 1505 days (IQR 188–2479). Stroke recurrence was found in 33 (7.4%) patients. Additional vascular events (stroke recurrence, myocardial infarction, heart failure, and peripheral occlusive disease) occurred in 71 patients (15.8%), as shown in Table 2. There were a total of 276 (61.6%) deaths during the follow-up period. Mortality rates were also significantly higher in VBC patients after a cut-off of 4 years.Further details are shown in Table 2. 

The median time to first stroke recurrence was 947 days (IQR 397-1497, range 46–2118 days). Only 8 cases of stroke (1.7%) occurred within the first 2 years. We further analyzed different variables for the cohort with recurrent stroke in comparison with patients in whom no stroke was observed. These findings are shown in Table 3.

**Figure 2:**
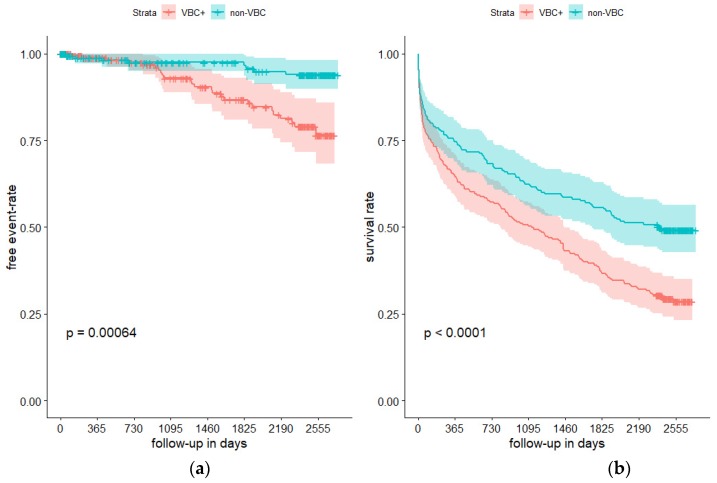
Survival analysis using Kaplan–Meier method of the presence of recurrent stroke ((**a**) Panel A) and death ((**b**) Panel B) during 7 years in the population of 448 patients with ischemic strokes according to presence of arterial calcifications in intracranial vertebral arteries and/or basilar artery. Vertical lines represent censored data. VBC+: vertebrobasilar calcifications present. Crosses represent censored (died and lost to follow-up) data.

Stroke recurrence in patients with VBC and nonVBC started to show a distinct pattern after 3-years of follow-up, as shown in Figure 2. In detail, after this period, patients with VBC had a significantly higher stroke recurrence rate than nonVBC patients. No-smoking history was more frequent in patients with recurrent stroke (60.6% vs. 45.8%, *P* = 0.017) as well as presence of VBC (75.8% vs. 52.5%, *P* = 0.011). Further distinct features between these two groups are shown in Table 3.

After excluding 47 patients (10.4%) who had died within 14 days from admission, the higher risk for patients with VBC for subsequent death, recurrent stroke, and vascular disease did not change (data not shown). Similarly, after exclusion of 125 patients (27.9%) with cardioembolic stroke at first hospitalization, there were no changes in associations beyond the aforementioned prognostic variables with outcome (data not shown). Stroke recurrence and death rates were more common in the presence of VBC (Figure 2).

**Figure 3:**
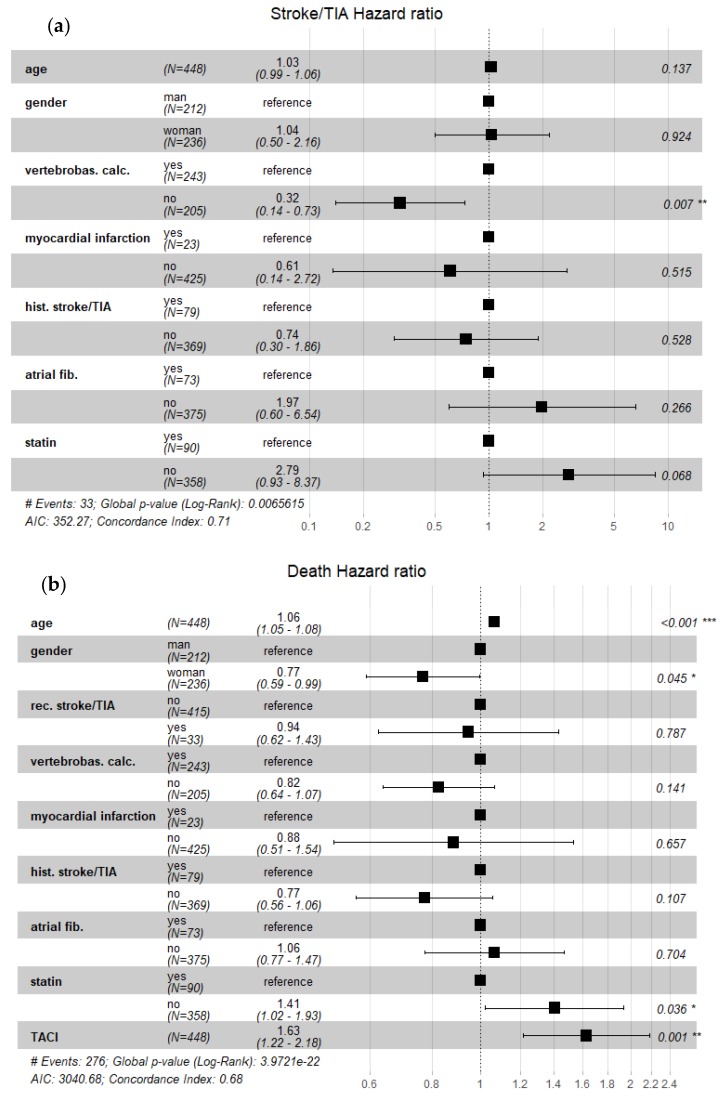
Forrest plots depicting hazard ratios for recurrent stroke/transitory ischemic attack ((**a**) Panel A) and death ((**b**) Panel B) in population of 448 ischemic stroke patients followed up to 7 years. Legends: vertebrobas. calc.–presence of vertebrobasilar calcifications; TIA–transitory ischemic attack; hist. stroke/TIA–history of stroke and/or TIA; rec. stroke/TIA–recurrent stroke/TIA; atrial fib.–history of atrial fibrillation; TACI–total anterior circulation stroke; and statin–taking statin upon inclusion in the study

For further analysis, only dichotomization (patients with VBC vs. nonVBC patients) was used since subgroup analysis would have been flawed due to small numbers (Figure 3).

**Figure 4:**
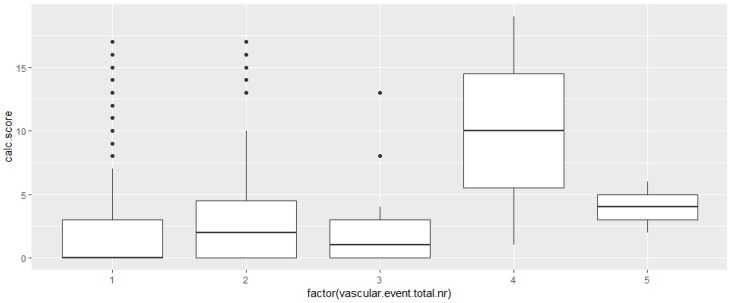
Severity of calcifications in vertebrobasilar arteries represented as calcium score (range 0–19) versus number of total vascular events on x-axis (prior stroke/TIA, index stroke, recurrent stroke/TIA, myocardial infarction, heart failure, peripheral arterial occlusive disease) in 448 patients hospitalized due to ischemic stroke and followed-up up to 7 years (Kruskal–Wallis *P* = 0.012).

Overall, the number of total vascular events (prior stroke/TIA, index stroke, and future vascular events) correlated with severity of VBC (corr. coefficient R 0.119, 95% CI: 0.026–0.209, *p* = 0.011, Kruskal–Wallis test *P* = 0.012, Figure 4).

### 3.4. Etiology of Recurrent Stroke

The etiology of recurrent stroke was reported in 16 (48.8%) cases and remained unchanged in comparison to the cause of the initial stroke in 9 patients (56%). The cause of stroke changed from LAA to another cause in 1 patient (6%), from CE to another cause in 1 (6%), from other cause to LAA in 1 (6%), and to SAO in 2 (12%). OCSP classification was reported in 14 patients with recurrent stroke and stayed the same in 5 (36%) patients, changed from TACI to PACI in 3 (21%), from PACI to TACI in 1 (7%), to LACI in 2 (14%), to POCI in 1 (7%), to UND in 1 (7%), and from LACI to PACI in 1 (7%). Changes of stroke localization as assessed by OCSP did not reach statistical significance.

### 3.5. Risk for Subsequent Vascular Events

We assessed the risk for stroke recurrence using a cox-proportional hazard model (Table 4 and Figure 3). The analysis showed that the presence of VBC was related to recurrent stroke with an adjusted hazard ratio (HR) of 3.13 (95% CI, 1.35–7.20; *P* = 0.007). Not taking a statin was marginally associated with stroke recurrence. We found an HR of 2.05 (95% CI, 1.21–3.47, *P* = 0.007) for any subsequent vascular event (stroke recurrence, myocardial infarction, heart failure, and peripheral arterial occlusive disease) during observation for the presence of VBC during the index event. Death of any cause was associated with age, male sex, not taking statins, and having TACI at first hospitalization (Table 4).

Adjusted for age, gender, presence of arterial calcifications in intracranial vertebral arteries and/or basilar artery, history of myocardial infarction, stroke, atrial fibrillation, and intake of statin. For death analysis, additional adjustment for total anterior circulation infarction (TACI) according to OCSP (Oxford Community Stroke Project classification) was made.

## 4. Discussion

This study disclosed that the presence of arterial calcifications in the vertebrobasilar territory, even after adjustment for potential confounders, is significantly associated with long-term risk of recurrent stroke, additional vascular events, and mortality after the occurrence of an ischemic stroke. Thus, this simple assessment of VBC on NECT at the index hospitalization could define a subgroup of patients that is at risk for further manifestation of cardio- and cerebrovascular disease and death. As VBC was not only associated with recurrent stroke but also the subsequent manifestation of additional vascular disorders, our findings raise the question whether there is a common and in addition preventable biochemical pathway for the progression of these conditions.

Our study corroborates and expands previous reports with regard to the relevance of intracranial calcification. It needs to be emphasized however, that our study focused on VBC, comprised a comparatively large number of patients, and also evaluated emergence of vascular disorders in addition to recurrent stroke. In contrast, Bugnicort et al. investigated calcifications in the vertebral, basilar, carotid, and middle cerebral arteries in 312 French patients with stroke and TIA [12]. Arterial calcification was detected in 83% of patients in that study with a mean follow-up period of 773 days vs. 54.2% and a median observation course of 1505 days in our study. They found an association of calcifications with “major clinical events” defined as death (vascular death, nonvascular death, and death of undetermined cause) or vascular ischemic events. The latter group included cerebral ischemic event (ischemic stroke/transient ischemic attack), cardiovascular ischemic event, and peripheral artery event. Yet, the population investigated differs from ours with regard to age. Indeed, that study recruited younger patients (67.0 ± 15.0 years vs. 76.0 (67.7–83.2)). Notably, patients without IAC (49.9 ± 16.0 years) were significantly younger than patients with IAC. They also had a higher rate of patients with new cerebrovascular events (32.8% vs. 7.4% in our study). This could be related to differences in the study population, genetic and acquired risk factors, but also more comprehensive diagnostic methods. Interestingly, in that study, patients experiencing some major clinical events almost all had IAC (97.0%) after the follow-up of 2 years. Furthermore, in that study there was no association of IAC with recurrent ischemic stroke [12].

A study by Lee et al. investigated 1017 Korean patients predominantly with ischemic stroke (92.3%) for presence of early vascular events defined as early progression or recurrence after primary ischemic stroke, coronary event, and vascular death within 2 weeks after index event [11]. Of note, the population was of Asian ancestry, which is known to have a significantly higher proportion of intracranial atherosclerosis and was also significantly younger than ours (60.7 ± 12.8 years) [19]. Indeed, they found a higher rate of IAC (61.4%) by studying both vessels of the anterior and posterior circulation. They used the same HU cut-off of 90 units for calcification detection as we did, whereas Bugnicort et al. used a threshold of 130 HU. Moreover, we excluded ICA calcifications from our analysis since those were present in more than 99% of our cohort. Although the definition used in this study cannot be extrapolated to our analysis, the Korean study showed a similar significant association of severe IAC for early major vascular events.

Strobl et al. evaluated the risk for future major cardiovascular events (MACE) with regard to intracranial arterial calcifications in 175 asymptomatic German patients. Mean age of the cohort was 78.3 ± 8.5 years and the mean follow-up was 39.8 ± 7.8 months [13]. Both carotid and vertebrobasilar artery calcifications were assessed and graded and summed to a calcium plaque score. MACE events occurred in 4.8% of patients, which was higher than in our study (15.8%), which is likely because we performed a case-control study following an index stroke event. Interestingly, they found that higher scores were related to a higher frequency of future vascular events, and no events were observed in patients without IAC.

A study from Turkey determined a potential association of VBC and the risk for stroke recurrence and functional outcome in patients with an index event defined as vertebrobasilar ischemic stroke. The cohort consisted of 188 patients with a mean age of 63.9 years [15]. The majority of patients had an SAO etiology (38%) and 79 (42%) harbored calcification in at least one vertebrobasilar territory. Similar to our sample, the rate of calcifications was lowest in the basilar artery. After 3-months of follow-up, 12 (6.6%) had recurrent stroke, which is significantly higher than in our study where only 3% had recurrent stroke within the first 3 months. The study could not find an association of VBC and recurrent stroke, which might be related to the short follow-up period.

Taken together, these studies cannot be compared directly due to different demographics and stroke etiology and variation in methodology and observational period. Yet, these studies grossly confirm the association of ICA/VBC and raised risk for subsequent cardio- and cerebrovascular disease.

Overall, the stroke recurrence rate of 7.4% is on the lower side. The etiology of recurrent stroke was assessed in the majority of them and changed in half of patients. On the contrary, stroke clinical classification and localization (assessed with OCSP) changed in almost 2/3 of patients, mainly from TACI to PACI. We could not confirm previous reports of association of cerebral artery calcifications and stroke localisation as we found that posterior ciruclation strokes were rare on recurrence (6.0% vs. 19.2% at index event) [20]. However, the study by Sohn et al. also reported numbers in a low range, so no definite conclusion could be drawn. Most probably, the presence of VBC should be taken as an overall indicator of atherosclerosis and not as a mediator of infarct location. Our observation is in concordance with previous reports who failed to establish an association between severity of IAC and cerebral infarct location [21,22].

Notably, the mortality rate in our cohort seems high, although a crude death rate approach reported rates of 77.0% after 8 years of follow-up in one recent study [23]. Although VBC was more frequent in women in our study, they had lower hazard for fatal outcome. Therefore, this could reflect the epidemiological findings that women, especially at this age (45–74) have a lower risk for stroke mortality than men [24]. This reflects the complex interplay between age, gender, and other risk factors contributing to mortality after stroke.

Presence of statin therapy at index hospitalization was associated with lower mortality therafter, and this finding is in the accordance with large meta-analysis showing lower mortality at 1 year after stroke [25]. Statins are routinely used in various stroke etiologies as secondary preventions due to its its lipid-lowering, vasculoprotective, and pleiotropic effects, which might be of benefit for patients with VBC [26]. Subclinical endothelial dysfunction is present in senescent population, a fact that we can indirectly confirm by finding VBC in significantly older patients [2].

Coronary artery calcification predicts which patients are at risk for fatal cardiac events but not the place of the plaque rupture [10]. This paradigm could be (conditionally) extrapolated to our population since we have not found significantly different stroke localization (i.e., posterior stroke) at index stroke or recurrent stroke, rather we found generally increased risk for future events.

There are two morphological manifestations of atherosclerosis–intimal (intima media) calcifications and medial (tunica media or internal elastic membrane) calcifications. Intimal calcifications are exemplified with focal calcifications and medial with crescent–circular calcifications. These patterns can be distinguished on NECT [10]. The secondary analysis from the randomized-controlled MR-CLEAN study showed that patients with medial calcification pattern when compared to patients with intimal calcification pattern benefit from endovascular intervention [27]. Although we did not assess calcifications patterns according to score proposed by Kockelkoren et al., and we were looking into vertebrobasilar arteries, we included morphological types of calcifications (crescent and/or circular). The proportion of VBC with medial pattern was lower than in ICA (35% vs. 56%). Furthermore, medial pattern cases were older and had higher triglyceride levels than those with focal calcifications. We did identify a higher rate of stroke recurrence with the presence of the medial calcification pattern, but it needs to be emphasized that there was a higher rate of mortality in patients with this arteriosclerosis subtype (23% vs. 12%, *P* = 0.003).

We acknowledge inherent study shortcomings. We did not assess for clinical severity at presentation or at discharge after the index stroke event. The mortality rate (rather expected due to the aging population in question and duration of follow-up) was high. The only comparable data from Slovenia are from Zvan et al., who reported a mortality rate of 63% but do not provide details about the follow-up period [28]. We did not assess for middle, anterior, and posterior cerebral artery calcifications since they are not prevalent in our population anyway. Although we have calculated the calcium score, we concluded that a subgroup-analysis would be biased due to the relatively small number of events. The evalation of cerebral vessels by CT or MR angiography was restricted to a small subgroup. This is of relevance since symptomatic intracranial arteriosclerotic stenoses have a high recurrence rate [29]. Moreover, a potential progression of calcifications upon stroke recurrence was not assessed. Furthermore, we did not have data on adherence to antithrombotic therapy. However, our population ascertainment was nearly complete since all patients of the district are referred to our hospital. Nevertheless, there is a possibility that some subtle clinical symptoms (minor stroke, TIA) or sudden death due to stroke outside the hospital were not recognised as strokes (or TIA) or were not transferred to the hospital and were therefore not recorded. Moreover, the post-hoc adjustement of a large number of variables bears the risk of model overfitting.

## 5. Conclusions

Our findings corroborate and extend the reports investigating associations of intracranial artery calcifications and vascular disease after an index cerebrovascular event. We did not detect associations with etiology or morphology of recurrent stroke, and therefore, we propose a more general effect of progressive vascular disease as the underlying process of our findings of recurrent vascular events.

## Figures and Tables

**Table 1:** Demographics, medical history, laboratory data, and stroke etiology of the 448 patients hospitalized due to ischemic stroke in the period from 9/2011 to 10/2012.

	Total (*N* = 448)	VBC (*N* = 243)	Without VBC (*N* = 205)	*P*
Man	212 (47.3)	103 (42.4)	109 (53.2)	0.029
Age	76.0 (67.7–83.2)	78.6 (71.0–84.1)	72.4 (63.4–79.9)	<0.001
Died in the study	276 (61.6)	172 (70.8)	104 (50.7)	<0.001
Medical history				
Stroke	79 (17.6)	53 (21.8)	26 (12.7)	0.013
TIA	24 (5.4)	16 (6.6)	8 (3.9)	0.292
Hypertension	323 (72.1)	185 (76.1)	138 (67.3)	0.045
Smoking history				0.013
Positive	148 (33.0)	66 (27.2)	82 (40.0)	
Negative	210 (46.9)	121 (49.8)	89 (43.4)	
Unknown	90 (20.1)	56 (23.0)	34 (16.6)	
Diabetes mellitus	93 (20.8)	51 (21.0)	42 (20.5)	0.908
Atrial fibrillation	73 (16.3)	49 (20.2)	24 (11.7)	0.020
Heart failure	67 (15.0)	43 (17.7)	24 (11.7)	0.085
Hyperlipidemia	98 (21.9)	58 (23.9)	40 (19.5)	0.302
Usage of statin drug	90 (20.1)	53 (21.8)	37 (18.0)	0.345
ICA Stenosis 50% or more	18 (4.0)	9 (3.7)	9 (4.4)	0.811
ICA treatment	5 (1.1)	4 (1.6)	1 (0.5)	0.381
Myocardial infarction	23 (5.1)	15 (6.2)	8 (3.9)	0.293
Peripheral arterial occlusive disease	23 (5.1)	12 (4.9)	11 (5.4)	0.834
Diagnostic procedures				
MRI	52 (11.6)	19 (7.8)	33 (16.1)	0.008
CT angiography	102 (22.8)	46 (18.9)	56 (27.3)	0.042
CT angiography of neck vessels	105 (23.4)	48 (19.8)	57 (27.8)	0.057
Ultrasound of neck vessels/intracranial ultrasound	169 (37.7)	85 (35.0)	84 (41.0)	0.204
ECG	447 (99.8)	243 (100.0)	204 (99.5)	0.478
24-h ECG	85 (19.0)	41 (16.9)	44 (21.5)	0.228
Heart ultrasound	125 (27.9)	57 (23.5)	68 (33.2)	0.026
Laboratory values				
Cholesterol (mmol/L)	4.8 (4.0–5.9)	4.8 (4.0–5.8)	4.8 (4.0–5.9)	0.886
Triglycerides (mmol/L)	1.4 (1.0–1.9)	1.5 (1.1–2.0)	1.3 (1.0–1.8)	0.171
LDL-C (mmol/L)	2.9 (2.3–3.8)	2.9 (2.3–3.7)	3.0 (2.3–3.8)	0.893
HDL-C (mmol/L)	1.2 (0.9–1.5)	1.2 (0.9–1.5)	1.2 (1.0–1.5)	0.375
Creatinine (mmol/L)	79.0 (65.0–98.0)	79.5 (65.0–99.8)	79.0 (65.0–98.0)	0.503
TOAST classification				0.017
LAA	90 (20.1)	43 (17.7)	47 (22.9)	
CE	125 (27.9)	78 (32.1)	47 (22.9)	
SAO	46 (10.3)	19 (7.8)	27 (13.2)	
Other	184 (41.1)	103 (42.4)	81 (39.5)	
UND	3 (0.7)	0 (0.0)	3 (1.5)	
OCSP stroke classification				0.002
TACI	85 (19.0)	46 (18.9)	39 (19.0)	
PACI	257 (57.4)	142 (58.4)	115 (56.1)	
LACI	17 (3.8)	2 (0.8)	15 (7.3)	
POCI	86 (19.2)	50 (20.6)	36 (17.6)	
UND	3 (0.7)	3 (1.2)	0 (0.0)	

Data are presented as number (percentages) or median (quintiles). Abbreviations: VBC: calcification in vertebrobasilar arteries; ICA: internal carotid artery; OCSP: Oxford Community Stroke Project classification; TIA: transient ischemic attack; TOAST: Trial of Org 10 172 in Acute Stroke Treatment; LAA: large artery atherosclerosis; CAE: cardioembolic; SAO: small artery occlusion; UND: undetermined; TACI: total anterior circulation infarct; PACI: partial anterior circulation infarct; LACI: lacunar cerebral infarct; POCI: posterior circulation infarct; UNK: unknown site of the infarct; LDL-C: indicates low-density lipoprotein cholesterol; and HDL-C: indicates high-density lipoprotein cholesterol.

**Table 2:** Clinical events after 7 years of follow-up.

	No. of Patients (%)
Cerebrovascular events	33
Stroke	29 (87.8%)
One rec. stroke	26 (78.8%)
TOAST	
LAA	5 (15.1%)
CE	1 (3.0%)
SAO	2 (6.0%)
Other	8 (24.2%)
UND	1 (3.0%)
OCSP	
TACI	3 (9.0%)
PACI	7 (2.1%)
LACI	2 (6.0%)
POCI	2 (6.0%)
UNK	3 (9.0%)
Two rec. strokes	2 (6.0%)
Three rec. strokes	1 (3.0%)
TIA	9 (2.0%)
Intracranial bleeding	1 (0.2%)
Other vascular events	*N* = 47
Myocardial infarction	10 (2.2%)
Heart failure	18 (4.0%)
Peripheral arterial occlusive disease	19 (4.2%)

Data are presented as number (percentages). Abbreviations: OCSP: Oxford Community Stroke Project classification; TIA: transient ischemic attack; TOAST: Trial of Org 10 172 in Acute Stroke Treatment; LAA: large artery atherosclerosis; CAE: cardioembolic; SAO: small artery occlusion; UND: undetermined; TACI: total anterior circulation infarct; PACI: partial anterior circulation infarct; LACI: lacunar cerebral infarct; POCI: posterior circulation infarct; and UNK: unknown site of the infarct.

**Table 3:** Demographic, historical, laboratory, and stroke etiological data of the 448 patients hospitalized due to ischemic stroke in the period 9/2011–10/2012 according to presence of recurrent stroke/transitory ischemic attack.

	Total *N* = 448	Recurrent Stroke (*N* = 33)	Without Recurrent Stroke (*N* = 415)	*P*
Men	212 (47.3)	15 (45.5)	197 (47.5)	0.858
Age	76.0 (67.7–83.2)	77.0 (68.6–83.3)	75.9 (67.6–83.1)	0.931
Died in the study	276 (61.6)	25 (75.8)	251 (60.5)	0.095
Medical history				
Stroke	79 (17.6)	6 (18.2)	73 (17.6)	1.000
TIA	24 (5.4)	0 (0.0)	24 (5.8)	0.242
Hypertension	323 (72.1)	26 (78.8)	297 (71.6)	0.427
Smoking history				0.017
Positive	148 (33.0)	12 (36.4)	136 (32.8)	
Negative	210 (46.9)	20 (60.6)	190 (45.8)	
Unknown	90 (20.1)	1 (3.0)	89 (21.4)	
Diabetes mellitus	93 (20.8)	6 (18.2)	87 (21.0)	0.826
Atrial fibrillation	73 (16.3)	3 (9.1)	70 (16.9)	0.330
Heart failure	67 (15.0)	6 (18.2)	61 (14.7)	0.611
Hyperlipidemia	98 (21.9)	5 (15.2)	93 (22.4)	0.390
Statin intake	90 (20.1)	4 (12.1)	86 (20.7)	0.365
ICA Stenosis 50% or more	18 (4.0)	1 (3.0)	17 (4.1)	1.000
ICA treatment	5 (1.1)	0 (0.0)	5 (1.2)	1.000
Myocardial infarction	23 (5.1)	2 (6.1)	21 (5.1)	0.683
Peripheral arterial occlusive disease	23 (5.1)	1 (3.0)	22 (5.3)	1.000
Diagnostic procedures				
MRI	52 (11.6)	3 (9.1)	49 (11.8)	0.784
CT angiography	102 (22.8)	7 (21.2)	95 (22.9)	1.000
CT angiography of neck vessels	105 (23.4)	7 (21.2)	98 (23.6)	0.834
Ultrasound of neck vessels/intracranial ultrasound	169 (37.7)	19 (57.6)	150 (36.1)	0.024
ECG	447 (99.8)	33 (100.0)	414 (99.8)	1.000
24-h ECG	85 (19.0)	8 (24.2)	77 (18.6)	0.487
Heart ultrasound	125 (27.9)	6 (18.2)	119 (28.7)	0.231
Laboratory values				
Cholesterol (mmol/L)	4.8 (4.0–5.9)	4.8 (3.9–5.7)	4.8 (4.0–5.9)	0.707
Triglycerides (mmol/L)	1.4 (1.0–1.9)	1.5 (1.3–2.0)	1.4 (1.0–1.9)	0.115
LDL-C (mmol/L)	2.9 (2.3-3.8)	2.8 (2.2–3.6)	3.0 (2.3–3.8)	0.686
HDL-C (mmol/L)	1.2 (0.9–1.5)	1.3 (1.0–1.6)	1.2 (0.9–1.5)	0.243
Creatinine (mmol/L)	79.0 (65.0–98.0)	77.0 (62.8–86.2)	79.0 (65.2–100.0)	0.225
TOAST classification				0.498
LAA	90 (20.1)	7 (21.2)	83 (20.0)	
CE	125 (27.9)	6 (18.2)	119 (28.7)	
SAO	46 (10.3)	2 (6.1)	44 (10.6)	
Other	184 (41.1)	18 (54.5)	166 (40.0)	
UND	3 (0.7)	0 (0.0)	3 (0.7)	
OCSP stroke classification				0.477
TACI	85 (19.0)	5 (15.2)	80 (19.3)	
PACI	257 (57.4)	20 (60.6)	237 (57.1)	
LACI	17 (3.8)	1 (3.0)	16 (3.9)	
POCI	86 (19.2)	6 (18.2)	80 (19.3)	
UNK	3 (0.7)	1 (3.0)	2 (0.5)	
Arterial calcifications in vertebrobasilar territory	243 (54.2)	25 (75.8)	218 (52.5)	0.011

Data are presented as number (percentages) or median (quintiles). Abbreviations: VBC: calcification in vertebrobasilar arteries; ICA: internal carotid artery; OCSP: Oxford Community Stroke Project classification; TIA: transient ischemic attack; TOAST: Trial of Org 10 172 in Acute Stroke Treatment; LAA: large artery atherosclerosis; CAE: cardioembolic; SAO: small artery occlusion; UND: undetermined; TACI: total anterior circulation infarct; PACI: partial anterior circulation infarct; LACI: lacunar cerebral infarct; POCI: posterior circulation infarct; UNK: unknown site of the infarct; LDL-C: indicates low-density lipoprotein cholesterol; HDL-C: indicates high-density lipoprotein cholesterol.

**Table 4:** Adjusted multivariable analysis using Cox proportional hazard model predicting stroke recurrence, any vascular event, and death in the cohort of 448 patients followed 7 years after first hospitalization due to ischemic stroke.

	Stroke Recurrence		Any Vascular Event		Death Due to Any Cause	
	HR (95% CI)	*P*	HR (95% CI)	*P*	HR (95% CI)	*P*
Age	1.03 (0.99–1.06)	0.137	1.01 (0.98–1.04)	0.342	1.05 (1.04–1.08)	<0.001
Woman	1.03 (0.49–2.16)	0.924	0.79 (0.47–1.32)	0.376	0.76 (0.59–0.99)	0.044
Arterial calcifications in vertebrobasilar territory	3.13 (1.35–7.20)	0.007	2.05 (1.21–3.47)	0.007	1.21 (0.93–1.57)	0.141
Prior myocardial infarction	1.65 (0.37–7.39)	0.514	1.45 (0.54–3.85)	0.451	1.13 (0.65–1.97)	0.657
Prior ischemic stroke	1.34 (0.53–3.37)	0.527	1.21 (0.64–2.28)	0.540	1.29 (0.94–1.77)	0.106
Diagnosis of atrial fibrillation	0.51 (0.15–1.67)	0.266	ß.97 (0.50–1.89)	0.937	0.93 (0.67–1.29)	0.703
Intake of statin at first hospitalization	0.35 (0.11–1.08)	0.068	0.82 (0.44-1.53)	0.547	0.71 (0.51–0.97)	0.035
Total anterior circulation stroke vs. other types					1.62 (1.21–2.17)	0.001
Recurrent stroke					0.94 (0.62–1.43)	0.787

Abbreviations: HR–hazard ratio, CI–confidence interval.
